# Patient-Specific Data Fusion Defines Prognostic Cancer Subtypes

**DOI:** 10.1371/journal.pcbi.1002227

**Published:** 2011-10-20

**Authors:** Yinyin Yuan, Richard S. Savage, Florian Markowetz

**Affiliations:** 1 Cambridge Research Institute, Cancer Research UK, Cambridge, United Kingdom; 2 Department of Oncology, University of Cambridge, Cambridge, United Kingdom; 3 Systems Biology Centre, University of Warwick, Coventry, United Kingdom; Accelrys, United States of America

## Abstract

Different data types can offer complementary perspectives on the same biological phenomenon. In cancer studies, for example, data on copy number alterations indicate losses and amplifications of genomic regions in tumours, while transcriptomic data point to the impact of genomic and environmental events on the internal wiring of the cell. Fusing different data provides a more comprehensive model of the cancer cell than that offered by any single type. However, biological signals in different patients exhibit diverse degrees of concordance due to cancer heterogeneity and inherent noise in the measurements. This is a particularly important issue in cancer subtype discovery, where personalised strategies to guide therapy are of vital importance. We present a nonparametric Bayesian model for discovering prognostic cancer subtypes by integrating gene expression and copy number variation data. Our model is constructed from a hierarchy of Dirichlet Processes and addresses three key challenges in data fusion: (i) To separate concordant from discordant signals, (ii) to select informative features, (iii) to estimate the number of disease subtypes. Concordance of signals is assessed individually for each patient, giving us an additional level of insight into the underlying disease structure. We exemplify the power of our model in prostate cancer and breast cancer and show that it outperforms competing methods. In the prostate cancer data, we identify an entirely new subtype with extremely poor survival outcome and show how other analyses fail to detect it. In the breast cancer data, we find subtypes with superior prognostic value by using the concordant results. These discoveries were crucially dependent on our model's ability to distinguish concordant and discordant signals within each patient sample, and would otherwise have been missed. We therefore demonstrate the importance of taking a patient-specific approach, using highly-flexible nonparametric Bayesian methods.

## Author Summary

The goal of personalised medicine is to develop accurate diagnostic tests that identify patients who can benefit from targeted therapies. To achieve this goal it is necessary to stratify cancer patients into homogeneous subtypes according to which molecular aberrations their tumours exhibit. Prominent approaches for subtype definition combine information from different molecular levels, for example data on DNA copy number changes with data on mRNA expression changes. This is called data fusion. We contribute to this field by proposing a unified model that fuses different data types, finds informative features and estimates the number of subtypes in the data. The main strength of our model comes from the fact that we assess for each patient whether the different data agree on a subtype or not. Competing methods combine the data without checking for concordance of signals. On a breast cancer and a prostate cancer data set we show that concordance of signals has strong influence on subtype definition and that our model allows to define prognostic subtypes that would have been missed otherwise.

## Introduction

Molecular data show great promise to stratify patients into distinct subgroups that are indicative of disease development, response to medication and overall survival prospects [1]. Such subgroups are highly useful in informing treatment decisions [2], [3]. Most current computational diagnostic approaches are based on gene expression data and cluster patients by co-expression of genes. For example, multivariate gene expression signatures have been shown to discriminate between disease subtypes, such as recurrent and non-recurrent cancer types or tumour progression stages [3]–[6].

In addition to expression data there are also many other data types that can be informative about a patient's disease status. For example, somatic copy number alterations provide good biomarkers for cancer subtype classification [7]. For this reason, the focus of research has recently shifted towards integrative clustering of complementary data types, e.g. [8]. The goal of integrative analysis is to identify clusters of samples that share not only expression profiles, but also other molecular characteristics such as copy number alterations. The subtypes of tumours identified in this way are more likely to share the same regulatory programs and underlying genomic alterations.

Data integration for subtype discovery poses several challenges that we address in this paper.

Challenge 1: Separating concordant from contradictory signals. While different molecular data are expected to share complementary information on common cellular processes, they can also contain contradictory signals because of the complexity of living cells and noise in the data. For example, genomic gains and losses may or may not be accompanied by concordant expression changes of the genes in the altered regions. The level of concordance may differ dramatically from patient to patient due to cancer heterogeneity. However, most existing integrative methods force different data types to be fused in all samples without reference to whether the data are concordant or contradictory in each patient.

Challenge 2: Selecting informative features. Identifying which measurements are informative about the underlying subtypes is particularly important when using genomic data because the number of measurements can be very large, e.g. in the tens of thousands or more in the case of microarrays. Because *a priori* we expect only a fraction of measurements to contain useful clustering information, extracting these features accurately will improve the quality and stability of clustering outcome. Additionally, identifying the relevant biological features can inform us about the underlying processes driving the disease.

Challenge 3: Estimating the number of subtypes. In many clustering algorithms this number is a parameter that needs to be set by the user [8]. Afterwards, the quality of the clusterings need to be compared, e.g. using stability indices [9]. However, jointly estimating the clusters together with their optimal number in a unified framework can improve results, because the most likely number of clusters can be inferred directly from the data.

These three challenges are not independent of each other: Whether or not the data show concordant signals for a subgroup of patients has a direct effect on which features should be selected as informative, which in turn has a direct influence on the estimate of the number of clusters. Thus, all three challenges need to be treated in an unified model.

Our approach is Patient-specific Data Fusion (PSDF) by Bayesian nonparametric modeling. In this paper, we propose a statistical model based on a two-level hierarchy of Dirichlet Process (infinite mixture) models (DPMs) [10], [11] that integrates copy number and expression data to jointly classify patients into cancer sub-groups. This model is an extension of the model presented in [12], modified to include a method of feature selection and adjusted to address a different problem with a number of advantages:

1. Different data types are fused (or not fused) on a sample-by-sample basis depending on the degree of concordance between two data types;
2. Input features are selected only if they are informative to clustering;
3. The most likely number of clusters are inferred automatically given the data.

Thus, the model not only identifies copy number alterations driving gene expression changes but simultaneously finds differences in regulation that distinguish one cancer subtype from the other. In doing so it explores the basic scientific question to which extend copy number data can be fused with expression data in integrative cancer studies.

everal integrative clustering approaches have been proposed in the literature [8], [13], [14]. A recent method is iCluster [8]. iCluster is based on a k-means approach that is extended to include more than one data type and performs feature selection in each data type independently. iCluster is fast and easily applied to more than two data types. However, compared to iCluster we have a more flexible mixture model underlying our own approach that in particular does not need the number of clusters (the ‘*k*’ in ‘*k-means*’) to be specified beforehand. In contrast to our model, iCluster assumes that both data are informative for all patients without checking for patient-specific consistency. In two case studies with cancer data sets [7], [15], we will show what impact these differences have and that our model compares favourably with iCluster in clinically important analysis results.

## Results

We introduce PSDF as an unified model to address the above three key challenges in patient subtype discovery. To demonstrate the power of this patient-specific integrative method, we analyse a breast cancer data set and a prostate cancer data set. High degree of concomitant changes has been observed in copy number and expression changes in breast cancer [15], [16]. In contrast, prostate cancer data display entirely different characteristics with relatively few co-ordinated genomic-transcriptomic changes [7], [17]. Therefore, these two cancer types represent two very different cases in terms of fusion ability, making them ideal for validating PSDF. Both the Matlab code for PSDF and pseudo-code for our work flow of data preprocessing and downstream analysis are available at https://sites.google.com/site/patientspecificdatafusion/.

### Patient-specific Data Fusion (PSDF) model

Bayesian nonparametric modeling provides a principled way to learn unknown structure in the data. Dirichlet Process (infinite mixture) models (DPMs) [10], [11] are Bayesian nonparametric models that have been widely used for clustering [18]–[25]. DPMs give us a sound interpretation of common cluster membership, that the data for those samples are drawn from the same underlying distribution. They also allow us to infer the most likely number of clusters given the data as part of the unified model.

PSDF groups patient samples on the basis of both gene expression and copy number alteration data. It also simultaneously distinguishes, on a sample-by-sample basis, between samples that can share concordant signal across the data types (**fused**) and those for which there is contradiction (**unfused**). We note that throughout this paper we will use the following terminology, relating to the concordance (or otherwise) of the two data sets for a given patient.

#### Fused

The patient sample belongs to one clustering partition, which is the same in both data sets. The clustering structure for this patient across the two data sets is said to be concordant.

#### Unfused

The patient sample belongs to different clustering partitions in each data set. The clustering structure for this patient across the two data sets is said to be contradictory.

By introducing a binary indicator parameter (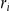, see the Methods section) for each sample, we can infer its fused/unfused state and because PSDF uses Markov Chain Monte Carlo (MCMC) sampling, this means we can determine for each sample the probability that it is fused (i.e. 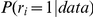).

By treating the data on a sample-by-sample basis, we can identify which samples are likely to belong in a fused state and which are likely to belong in an unfused state. This gives us a principled way of finding subgroups of samples with concordant or heterogeneous structure which, as we show below, leads to new insights about the disease and its subtypes.

Feature selection (biomarker discovery) is also built-in to PSDF, using two sets of binary indicator parameters, 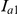 and 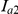. These switch off/on features in each data set, so we can infer as part of the modelling process which features are contributing to the analysis. Again, because PSDF uses MCMC sampling, this allows us to determine 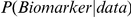 for each feature, the probability that it is an informative biomarker in the analysis. This both improves the quality of the subtypes by discarding “noisy” features, plus allows us to identify which features in the data are biologically informative and may hence be biomarkers for the disease.

Fuller details on this can be found in the Methods section.

### Case study 1: Fusion clusters reveal prognostic breast cancer subtypes

The breast cancer data from [15] contains both copy number and expression data for 106 tumour samples, with 26,755 copy number probes and 37,411 expression probes. Even for a clustering method with feature selection capability, it is convenient to remove the mostly obviously uninformative “noise” features. To preselect features with functional implications in a principled, controlled manner, we take the following steps.

First, copy number data are filtered based on whether there is a concomitant change between a locus's copy number and its own expression. This is to exclude passenger events without explicit downstream effects. Each expression probe is matched to its nearest copy number probe allowing for multiple matches, i.e. a copy number probe can be matched to multiple expression probe. This resultes in 37,411 matched pairs of copy number and expression data annotated by expression probes. We then calculate the adjusted 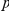-values of the correlations of each pairs of copy number and expression probes, and a copy number probe is selected if the corresponding 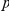-value is smaller than 0.1. Still there are highly similar copy number profiles among the selected copy number probes. To remove redundancy, copy number data of the selected probes are then merged based on their similarity using CGHregions [26], which results in 379 regions. Finally, both of the copy number signatures from the merged regions and all expression profiles passing the above 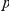-value threshold are ranked by the Wald test in predicting breast-cancer-specific survivals. The best 200 of each type of data are used for clustering.

#### Distinguishing concordant from contradictory signal

**Figure 1:**
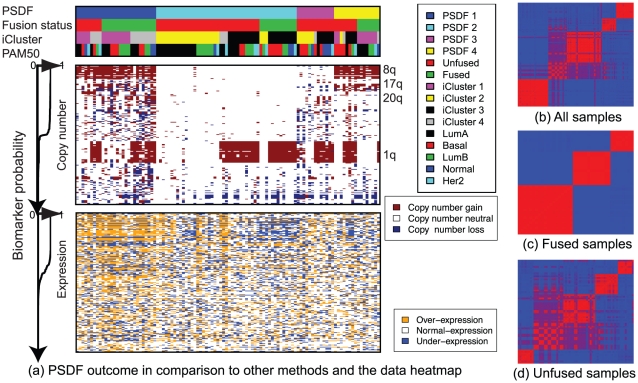
(a) Breast cancer data heatmap sorted by PSDF outcome compared with another integration method iCluster, and the PAM50 subtypes based on expression alone. Features are ranked by their probability of uses in the MCMC sampling from high to low respectively for copy number and expression features, as indicated on the left. (b–d) Posterior similarity matrices (red: high posterior probability between patient samples; blue: low posterior probability).

PSDF yields 4 clusters for all 106 breast cancer samples and 3 fused clusters, containing only samples for which 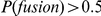. We then use 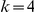 as input to iCluster to obtain the iCluster partition. These results, together with the PAM50 partition as a popular breast cancer subtype classification in the literature generated using the breast cancer gene expression signatures in [2], are shown together with the input data in Fig. 1(a). Fig. 1(b–d) show the posterior probability matrices of two given samples being in the same cluster. The posterior is averaged over both data sets.

The case study results show the power of patient-specific data fusion. The similarity matrix for all items (Fig. 1(b)) shows that Cluster 2 has some levels of substructure. From the heatmap in Fig. 1(a), the expression features have distinctly different value for that cluster, while the copy number are primarily neutral. This is the reason why only part of this cluster is fused by both data. The fused samples in this cluster, as shown by its simlarity matrix in Fig. 1(c), have well defined structure, indicating that the data are fused by concordant features from two data types.

The unfused samples are also interesting. Part of Cluster 1, 2, and 4, as well as the entire Cluster 3 are unfused, for which lots of ambiguity exists in the similarity matrix (Fig. 1(d)). The unfused samples in these clusters, although having similar copy number alterations, are with a range of different expression values, suggesting that there may be insufficient gene expression signal-to-noise for those samples to fuse. These samples are good examples of a case where the two data sources should not be forced to fuse, because part of the signals are contradictory.

The case study results also demonstrate the power of feature selection. For the informative features selected by PSDF, there are 60% of copy number and 40% of expression features. Copy number features from 8q (Chromosome 8 q arm), 17p (Chromosome 17 p arm), 17q, 20q are among the most frequently used. These regions harbor some of the most well known genes in breast cancer. For example, 8q contains *MYC*, 17q has *BRCA1*, 17p encodes *TP53*, and 20q harbors *NCOA3*. Interestingly, 1q features are not selected by our model but iCluster. This is likely to be due to the low concordance between the copy number alterations of this region and the expression features.

#### Prognostic breast cancer subtype discovery

**Figure 2:**
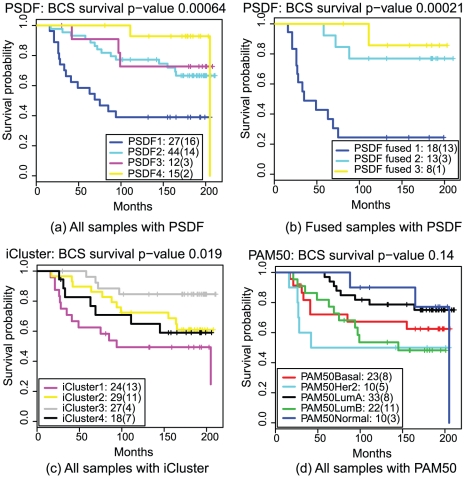
Kaplan-Meier survival curves of PSDF, iCluster, and PAM50 results with their 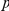
 **-values (log-rank test) for breast cancer specific survivals.**

Clinical follow-up for this data set facilitates the assessment of data-driven subtype discovery with respects to their prognostic outcome. For PSDF, the Kaplan-Meier breast cancer specific survival curves for all samples reveal a low survival group (PSDF 1), a good outcome group (PSDF 4), and two intermediate groups (PSDF2 and 3), as shown in Fig. 2(a). Log-rank 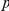-value shows test result of the null hypothesis that each cluster in the partition is drawn from the same underlying survival distribution. The same are plotted for the fused samples from PSDF, iCluster and PAM50 results (Fig. 2(c)). The 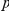-value for PSDF is much lower than the other two. It also has a group with significantly worse outcomes (the dark blue group) which is bigger and contains more events (deaths) than the worst group from iCluster (purple). Another interesting observation is that PSDF partition is able to separate early events (PSDF 1) from late events (PSDF 2, 3, 4), while these events are mixed up among the iCluster groups.

Fused subtypes are prognostic in both events and timing. For the three fused clusters in Fig. 2(b), the poor outcome fused group has only 18 members but 13 deaths in the early stage (16–69 months), while PSDF fused 2 has events from 5 to 88 months and PSDF fused 3 with only 1 at 111 months. The iCluster partition for these fused samples do not exhibit such behaviour. This may suggest that the concordant copy number and expression changes may help predict both events and their timing.

Subtype-specific features reveal functional implications. With respect to the genetic features that characterise these subtypes, the poor prognosis subtype (dark blue) has 8q copy number gains and over-expressions (see Fig. 1). Meanwhile, the good outcome group (yellow), although also has 8q gains, do not have the over-expressions. This implies that the combination of copy number gain together with functional over-expressions can be associated with increased risk in breast cancer. Since these subtypes are defined by these genetic features and their functions are likely to be linked to the disease outcome, we further explore the functional implication of the unique features for each subtype.

For each of the cluster/subtype, we extract its cluster/subtype-specific genes based on both copy number and expression data. Limma [27] is used to score all genes on the microarray by comparing the expressions or copy number data in a cluster with the rest. As a result, genes with significantly differential copy number or expression changes are assigned a low 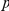-value (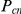 or 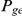). Log fold change score for copy number 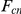 or expression 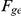 is also computed. A gene's copy number change or expression change is termed subtype-specific if the corresponding 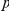-value are smaller than 0.1 and absolute log fold change larger than 0.2. This enables detection of genes associated with a specific cancer subtype on either the genomic or transcriptomic level. With the subtype-specific genes, we can then explore the functional implications of the genetic alterations associated with a particular cancer subtype. We are particularly interested in the poor outcome groups from our model (dark blue and purple) and focus on these two subtypes in the subsequent analysis.

#### Subtype-specific network modules

The subtype-specific genes are combined with a Protein-Protein Interaction (PPI) network to extract functional network modules. The PPI network is downloaded from HPRD, release 9, April 2010 [28]. The R package BioNet [29] can extract an optimal network module with highest overall node scores, which, in this case, are the Limma 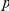-values for the subtype-specific genes.

**Figure 3:**
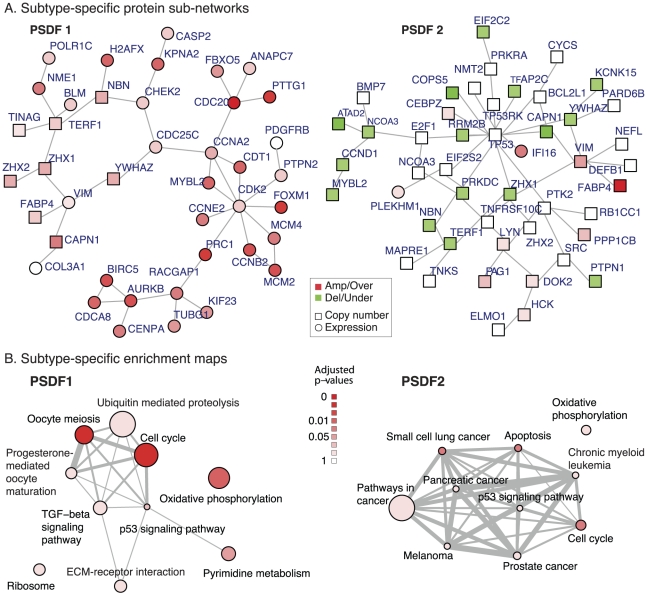
Network modules and enrichment maps as part of the functional follow-up analysis for the breast cancer subtypes: (A) Subtype-specific network modules for PSDF 1 and 2. The node color in the network modules indicates the type of alterations relative to this cluster: red - copy number gain or over-expression, green - copy number loss or under-expression. The shape of nodes indicates the type of data: square - copy number, round - expression. (B) the KEGG pathway enrichment maps for PSDF 1 and 2. The node colors indicate the significance of enrichment result and the thickness of the edges indicates the amount of overlaps between pathways.

The network module of PSDF 1 in Fig. 3(A) is characterised with the over-expressions of cyclin genes such as *CCNE2*, *CCNB2*, *CCNA2*, *CDC25C*, *CDC20*, as well as copy number gains of several genes on Chromosome 8. The connection between the poor outcome and over-expression of cyclin genes is in line with the literature, some of which are known prognostic markers in breast cancer [30], [31]. The functional interactions between subtype-specific genes are also interesting, for example, *CHEK2* checkpoint homolog is a putative tumour suppressor. When activated, the encoded protein is known to inhibit *CDC25C* phosphatase, preventing entry into mitosis, and has been shown to stabilize the tumour suppressor protein p53, leading to cell cycle arrest in G1.

The subtype-specific module 2 in Fig. 3(A) is featured with predominantly copy number losses of genes centering at *TP53*. *TP53* is an important tumour suppressor and marker in breast cancer [32]. Its protein product p53 regulates a large number of genes that control a number of key tumour suppressing functions such as cell cycle arrest, DNA repair, senescence and apoptosis. This module also features relatively low copy number of several important genes in cancer such as *NCOA3*, a nuclear receptor co-activator that interacts with nuclear hormone receptors to enhance their transcriptional activator functions, and *CCND1* whose copy number gain and over-expression can alter cell cycle progression and may contribute to tumorigenesis, as well as *MYBL2* which has been shown to activate the cell division cycle 2, cyclin D1.

#### Subtype-specific KEGG pathways

Meanwhile, KEGG [33] pathway enrichment analysis can be applied to the top 800 subtype-specific genes for the discovery of subtype-specific signaling pathways as potential targets for treatment. We use the enrichment map [34] in R package HTSanalyzer [35] for visualizing the functional enrichment of the two subtypes associated with poor prognosis. Using a hypergeometric test on the subtype-specific genes, we search for deregulated KEGG pathways specific to a given cancer subtype. The pathway maps in Fig. 3(B) show the enriched pathways in the two PSDF subtypes with an adjusted 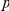-value cutoff at 0.05. The node color indicates the significance by the hypergeometric test 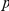-value, and edge widths corresponding to the amount of overlaps between pathways.

The PSDF-specific pathways for PSDF 1 include Cell Cycle, Oxidative Phosphorylation, Pyrimidine metabolism, which are known to be deregulated in breast cancer [36], [37]. It also further supports that the cyclin over-expression module of this subtype is the functional component in this subtype. We noted before that the gain of the same genomic region without over-expression in PSDF 4 corresponds to a favorable outcome. This module is actively involved in the signaling pathway and likely to be the key to this subtype.

PSDF 2 is characterised by deregulations in the Apoptosis pathway which includes several important genes such as TP53. Combined with the network module in Fig. 3(A), the pathway analysis result leads to the conclusion that this subtype is featured with genes losses centered at *TP53* in the Apoptosis pathway. Therefore, while over-expression of the Cell Cycle pathway points to early stage breast cancer deaths in the worst outcome subtype, copy number loss of p53 signaling pathway characterises the subtype with intermedia survival outcome.

### Case study 2: New prostate cancer subtype of very poor survival outcome

For the prostate cancer data set, there are 150 tumour samples with both copy number and expression data [7]. The expression data were profiled with Affymatrix Human Exon 1.0 ST array which contains 229,581 probes after quality filtering. For the copy number data, there are 43,416 probes on Agilent 244K array comparative genomic hybridization array.

To extract features, we use a slightly different approach since the scale of this data set is much larger than that of the breast cancer data. Substantially larger number of probes compared to the breast cancer study means that the probe-centric method is not suitable, hence we take a gene-centric method by aggregating copy number and expression data to 12,718 genes based on array annotation. For copy number data, the aggregation is done by taking the median for probes within a gene. For the expression, the probe most highly correlated with the copy number profile of a gene is chosen to represent this gene. Even if so, only modest correlations are observed between the two data types. Finally, 286 genes with highly correlated copy number and expression (adjusted 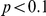) from the two data sets are used as clustering input.

#### Prognostic prostate cancer subtype discovery

**Figure 4:**
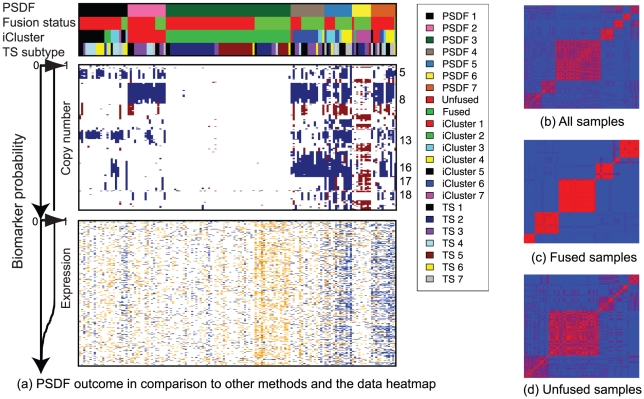
(a) Prostate cancer data heatmap sorted by PSDF outcome comparing with another integrative clusteringmethod iCluster and the TS subtypes based on copy number data alone. Features are ranked by their probability of uses in the MCMC sampling from high to low respectively for copy number and expression features, as indicated on the left. Color codes for the heatmap are the same as in Fig.1(b–d) Posterior similarity matrices (red: high posterior; blue: low posterior).

**Figure 5:**
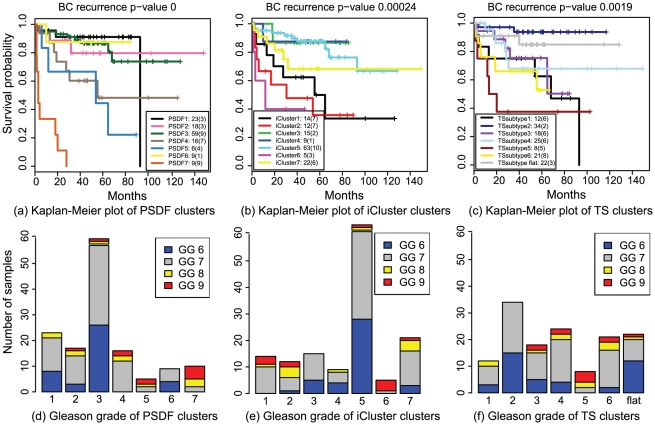
Comparison of prostate cancer data clustering result from our method to that from iCluster and TS subtypes using survival curves and 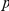
 **-values (log-rank test) for biochemical recurrence, as well as the distribution of Gleason grade (GG) as an important prognostic factor of prostate cancer.**

To compare with PSDF outcome, we take the original subtype classification for this data set [7], referred to as “TS subtype”, and the iCluster outcome. Previously, seven subtypes (Cluster 1–6 and a “flat” cluster [7]) were found based on unsupervised hierarchical clustering using copy number data alone as the authors found that the expression data seem to have weaker prediction power for biochemical recurrence. Interestingly, without prior knowledge of cluster numbers, PSDF also yields seven clusters, supporting that there are seven distinct subtypes in the data. All copy number features were selected as well as a subset of expression features as indicated by the biomarker probability curves in Fig. 4, supporting the findings in [7] that copy number data are more informative in prostate cancer. To enable fair comparison, we use iCluster to obtain a seven-cluster outcome. All different clustering and the input features are visualised in Fig. 4. Their Kaplan-Meier curves for biochemical recurrence and the distributions of Gleason grade are plotted in Fig. 5.

Significant differences of recurrence outcome was found among the PSDF clusters (log-rank test 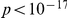), which can be categorised to three outcome categories: poor outcome (PSDF 7), moderate (PSDF 4 and 5), and good (PSDF 1, 2, 3, and 6). Strikingly, a unique cluster to the PSDF clusters is the poor outcome cluster PSDF 7 which contains 9 patients all with recurrences. Like with the breast cancer case, this poor outcome cluster contains mainly early-stage recurrences, all of which occur before 30 months of diagnosis, highlighting its aggressiveness. It is worth noting that this cluster persists even when we run PSDF with a different set of features (data not shown), indicating its robustness. With respects to the Gleason grade, this worst outcome group is larger than those of the other two clustering outcome (Fig. 5(d–f)). Notably, this group contains a mixture of grade 7, 8 and 9 tumours but all with early deaths, suggesting that PSDF might captures information missed by the Gleason grade.

Interestingly, although PSDF and iCluster share two clusters, PSDF/iCluster 2 and 3, this poor outcome cluster PSDF 7 is lost among the iCluster clusters. PSDF 7 is also not identified by the original TS subtypes. This is because if only copy number data are used, PSDF 4 and PSDF 7 would be clustered together. If only expression data are used, PSDF 5 and PSDF 7 are likely to be jointed. Thus, clustering on a single data type is not able to recover this subtype, highlighting the strength of data fusion. Additionally, integrative clustering methods that force all samples to be fused, such as iCluster, will tend not to recover PSDF 7, instead dividing those samples between PSDF-4- and PSDF-5-like clusters. This is evidenced by that fact that PSDF 7 is largely unfused (Fusion status in Fig. 4(a)). Hence, taking a patient-specific approach here is vital to discovering this poor outcome group, again supporting the importance of distinguishing between concordant and discordant signals in subset of samples.

#### Subtype-specific network modules and their pathways

**Figure 6:**
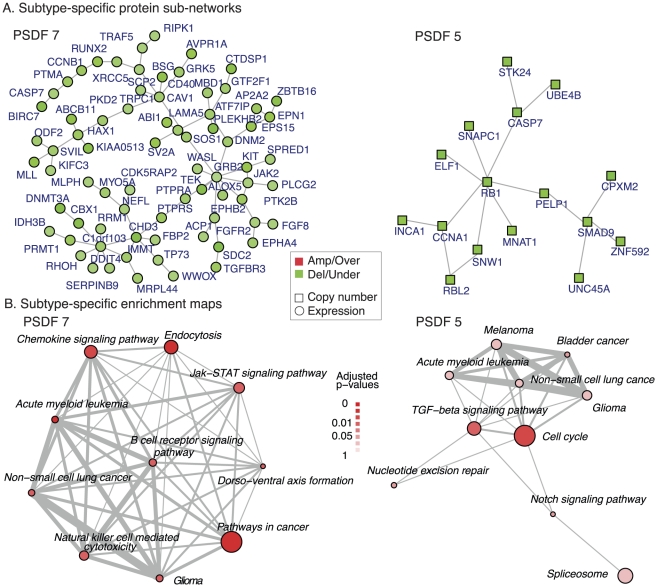
Prostate cancer subtype-specific network modules and enrichment maps: (a–b) Subtype-specific network modules for PSDF 7 and 5. The node color in the network modules indicates the type of alterations relative to this cluster: red - copy number gain or over-expression, green - copy number loss or under-expression. The shape of nodes indicates the type of data: square - copy number, round - expression. (c–d) KEGG pathway enrichment maps for PSDF 7 and 5 module genes. The node colors indicate the enrichment significance and the thickness of the edges indicates the amount of overlaps between pathways.

We focus on the two worst outcome groups PSDF 7 and PSDF 5 and examine their subtype-specific genes in the same manner as done before for the breast cancer data set. Interestingly, PSDF 7 is characterised by the under-expression of many functionally-related growth factors, such as *GRB2* and *FGFR2*, as well as cancer-generic genes such as cyclin *CCNB1*, hypothesized tumour suppressor *TP73* and mixed-lineage leukemia *MLL*. The enrichment map of PSDF 7 in Fig. 6(B) shows that its subtype-specific genes are enriched with many cancer pathways, among which the most significant are Chemokine signaling pathway and Endocytosis. Studies on chemokine signaling pathways not only confirm their roles in regulating immune responses [38], but also suggest that chemokines are critical for cancer progression with their impacts on the tumor microenvironment [39]. There are increasing evidences that endocytosis plays a central role in control of the cell cycle, mitosis, apoptosis and cell fate determination, which projects to hyper-proliferative conditions like cancer [40], [41]. In keeping to these studies, our results here collectively suggest the contribution of down-regulation of these pathways to poor clinical outcome in prostate cancer.

On the other hand, PSDF 5 features copy number losses of the functional network module centered at *RB1*, a negative regulator of the cell cycle and a tumor suppressor. *RB1* encodes a protein which stabilises constitutive heterochromatin to maintain the overall chromatin structure. The active, hypophosphorylated form of the protein binds transcription factor *E2F1* which may induce suppression of apoptosis in prostate cancer [42]. Hence copy number mutations in *RB1* may lead to large-scale transcriptional deregulations. Other genes in this module include cell cycle gene *CCNA1*, Nuclear receptor coactivator *SNW1*, and *CASP7*. *CASP7* encodes a protein in the caspase family, which plays a central role in the execution-phase of cell apoptosis. *CCNA1* was found to bind to important cell cycle regulators, such as RB family proteins, transcription factor *E2F1*, and the p21 family proteins. With only 16 genes, the network module of PSDF5 is enriched with Cell cycle and TGF-beta signaling pathway genes (Fig. 6(d)). DNA copy number losses of many important genes in these pathways indicate the potential roles of these genes in this cancer subtype.

## Discussion

This paper explores the potential of patient-specific data fusion to enhance prediction power in cancer subtype discovery. Cancer subtype discovery combining both genomics and transcriptomics leads to a more comprehensive understanding of the heterogenous cellular contexts. By using a flexible, nonparametric model such as the model presented in this paper, we can learn both the concordant and contradictory structures underlying those multiple data types. This structure leads to an improved understanding of the functional components and pathway regulations for each cancer subtype, something that is essential for the future development of targeted therapeutics. **Our contributions are therefore as follows**.

- We propose a model that is able to separate concordant and discordant signals and find sub-structures based on either one data type or both. This is in contrast to most previous approaches, where samples are typically forced to cluster together based on both data types
- We demonstrate that by identifying the concordant/*fused* or otherwise/*unfused* samples, we can identify cancer subtypes that give superior prognostic value for both outcome and time to events/death
- Functional analysis on subtype-specific genes reveals the genetic components that may lead to the poor outcome cancer subtypes. These are worthy of future investigation and may lead to therapeutic benefits.

With both breast cancer and prostate cancer data, PSDF is able to discover poor outcome subtypes with early-stage, highly frequent recurrences/deaths. These subtypes are not identified by other methods which either force to fuse data on all samples, or cluster patients based on single data type. We show that there exist both concordant and contradictory signals in these data, which, when forced to cluster together, can result in inferior subtype identification. Moreover, data fusion is necessary in predicting both events and timing of cancer survivals/recurrrences. Hence, taking this approach is vital in the discovery of new disease subtype consisting of early-stage events.

A promising aspect of studying cancer subtypes is the identification of key pathways altered unique to this subtype. Our network analyses show functionally interacting genes in the subtype-specific network modules whose deregulations may contribute to the poor outcome of a cancer subtype. The pathway enrichment analysis facilitates functional interpretation of the new clusters/subtypes in a coherent manner with the network modules. Under-lying driver events for poor outcome may be revealed during this process, such as the over-expression of the Cell Cycle pathway in breast cancer, and the under-expression of Endocytosis and Chemokine signaling pathway in prostate cancer. Further exploration of these results may lead to the discovery of new genes participating in the cancer-related pathways, as well as the identification of treatment target and the development of pathway inhibitors.

Our analysis results also highlight the difference between different cancer types. Previously, relatively low concordance between prostate cancer copy number and expression has been reported [17], in contrast to the high-level correlations generally observed in breast cancer. In addition, unlike breast cancer where RNA expression are predictive of recurrence, copy number changes in prostate cancer have been found to outperform expression in prediction [7]. Different degrees of concordance in the data lead to significantly different clustering results – while fused clusters in highly concordant breast cancer data are prognostic, an unfused subtype in prostate cancer turns out to be extremely aggressive. The results from the breast and prostate cancer data sets are in fact strong statements that different cancer types should be treated differently by statistical methods. Hence, a versatile tool such as PSDF is particularly suitable for this field.

## Methods

PSDF extends the model of [12] to include feature selection. The model is motivated by the need to address three main challenges in data-fusion-based clustering, namely (i) to separate concordant from contradictory signals, (ii) to identify which features are informative and (iii) to estimate the number of disease subtypes.

**Figure 7:**
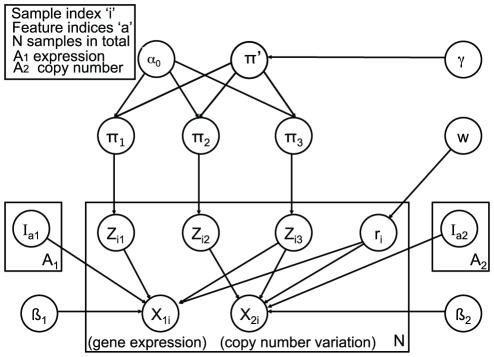
Graphical representation of the PSDF model presented in this paper. The 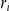 indicator variables allow the model to perform data fusion on a sample-by-sample basis, defining the states *fused* (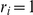) and *unfused* (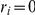). The prior probability of fusion is defined by 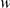 and is set in all cases to 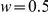 for the results in this paper. The 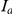 parameters are binary switches that select individual features in each data set. The number of clusters is given by the number of unique values assigned to the 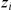 variables, which denote cluster membership in a given context. The 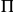 parameters are mixture weights for the Dirichlet Processes and are marginalised analytically. 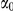 and 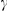 are concentration hyperparameters for the Dirichlet Processes and are sampled as part of the MCMC procedure.

PSDF is constructed from a two-level hierarchy of Dirichlet Processes, as shown in Fig. 7. Each patient has a binary state (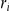) that defines whether their data are concordant across the data sets, either fused (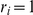) or unfused (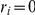).

Within any given mixture component from the Dirichlet Processes, we model the (discretised) data as being drawn from a multinomial distribution with a weakly informative multinomial prior. The features are assumed to be independent, giving rise to a naive Bayes data model for each data set. We use this data model for both gene expression and copy number data sets. Since our method use discretised data as input, copy number calls are made with R package CGHcall [43]. Without match normal expression data, we use quantile discretisation to deem the top 10% log2 ratio data as over-expressions and bottom 10% data as under-expressions, similar to [44], [45]. In cases when match normals are available, appropriate methods such as the one in [46] can be used for discretising the expression data. As a result, the copy number data are discretised into three levels of loss, neutral, and gain, and the expression data are discretised into three levels corresponding to under-, normally- and over-expressed.

We note that in principle, this model could be extended to 3+ data sources. In practice however, this will become unwieldy, and so we restrict ourselves in this paper to considering fusion between two data sources. We are currently developing a related model that will scale much better with increasing numbers of data sources.

### Feature selection

The naive Bayes data model used in [12] models data for a given feature as being drawn from a multinomial distribution with unknown class probabilities. Choosing a conjugate (Dirichlet) prior, these unknown class probabilities can be marginalised out to give a marginal likelihood for each feature in each cluster.
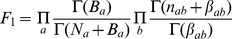
(1)Where 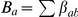 and 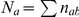, 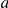 is the index over features and 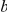 is the index over discrete data values. The 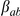 are the Dirchlet prior hyperparameters, which in this case are set to match the known proportions of each data value in the data set (which is prior knowledge here, as we define the data discretisation). These proportions are scaled to sum to 1.5, which is the sum of the Jeffreys' value (0.5) over the three possible data values, hence representing only a weakly-informative constraint.

To perform feature selection, we will consider two different likelihoods for a given feature, corresponding to the feature being *off/on*, as denoted by an indicator variable 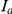. For 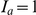, we simply use the multinomial-Dirichlet marginal likelihood, as before. For 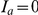, we fix the class probabilities to the expected prior values, given the spread of discrete input values for the given feature.
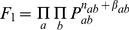
(2)Where again 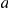 is the index over features and 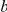 is the index over discrete data values. The 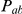 are simply taken as the proportion of each data value in a given feature across the whole data set, with a minimum count of one assigned to each data value.
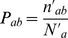
(3)Where 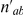 and 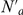 are required to have minimum of one count per class.

This has the effect of defining an ‘indifference’ likelihood, where it makes no difference to the overall posterior (for the given feature) to which cluster any given sample is assigned. It is straightforward to write down the conditional distribution for a single indicator variable 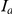, so we Gibbs sample each in turn when producing a new MCMC sample.

The switching on/off of a given feature can be regarded as a kind of model selection. Considering the limit of many samples (and hence negligible uncertainty in the value of the class probabilities for 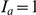), the ‘indifference’ likelihood is simply the expected case if the samples are randomly assigned to clusters. For finite numbers of samples, the ‘indifference’ likelihood is inherently simpler (in the sense that the class probabilities are known), so the feature selection becomes a competition between this simplicity and the greater ability of the 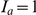 case to explain non-random cluster assignments.

### MCMC performance

To give improved mixing, we run 50 MCMC chains for each analysis. The chains are 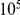 samples long, with the first 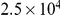 removed as a burn-in. The remainder are sparse-sampled by a factor of 10 for computational convenience and then used to produce the outputs.

All chains are examined using the R package *CODA*. In particular, the time-series and histograms for each parameter/chain pair are examined by eye for any obvious anomolies that would indicate incomplete mixing.

The multiple MCMC chains are used to compute uncertainties in statistics of interest (for example, the probability that a given feature is selected). This gives us a direct measure of chain mixing quality.

Each chain runs to completion in less than 48 hours on nodes of the University of Warwick's high performance computer cluster.

### Simulation study

In order to validate our model, we performed a simulation study. We constructed a pair of synthetic data sets. For each synthetic data set, we started with the 106 *signal items* and 200 *signal features* in the copy number variation data from [15] (which is also analysed in Section. These items will therefore (by construction) be fused as they share identical clustering structure across the two synthetic data sets. We note that this is a reasonable test of the method because in the real analyses both copy number and gene expression data sets are discretised into three levels. These synthetic data represent a good way of constructing items that share concordant signals across the two data sets.

To each synthetic data set, we then added 50 *noise items*. These items are drawn by replacement from the signal items and are drawn separately for each synthetic data set. For example, a given noise item may be a copy of signal item 15 in the first synthetic data set, and signal item 59 in the second synthetic data set. These noise items are therefore drawn from the existing clustering structure of each synthetic data set, but in general they will not be fused (excepting the case where by coincidence they are both drawn from the same underlying cluster). This then gives us 156 items in total.

Finally, we added to each synthetic data set 200 *noise features*. The data for these features are drawn with replacement from the original data. Therefore, while they reflect the distribution of data values in the signal features, they are entirely random and without clustering structure. As such, we expect them o be rejected by feature selection.

Table 1 shows the results of an analysis of these synthetic data. The method successfully rejects all 400 noise features across the two data sets. 8 signal features are also rejected at this level, but we note that some level of feature rejection is expected of signal features, as some of them will be uninformative.

**Table 1:** Results from the simulation study.

	signal items	noise items
fused	105	17
unfused	1	33

	signal features	noise features
selected	392	0
rejected	8	400

Shown are the fused/unfused items (top) and the selected/rejected features (bottom). The fusion threshold is set at 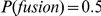 and features are rejected if 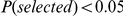. We note that some level of feature rejection is expected of signal features, as some of them will be uninformative. In spite of this, the separation of signal/noise features is close to perfect.

The method successfully finds 105 of the 106 fused items. It also identifies 17 of the noise items as being fused. We note that we expect some level of coincidental fusion for the noise items, where they happen to have been drawn from the same cluster. For example, if we assume there are 5 (equally-sized) underlying clusters in the copy number data, we expect 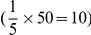 coincidentally fused noise items. We note that here, 25 MCMC chains of length 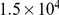 samples are sufficient to achieve reasonable convergence. We conclude that our method performs well in identifying both fused/unfused items and selecting appropriate features in each data set.

## References

[1] Perou CM, Børresen-Dale AL (2010). Systems biology and genomics of breast cancer.. Cold Spring Harb Perspect Biol.

[2] Sorlie T, Tibshirani R, Parker J, Hastie T, Marron JS (2003). Repeated observation of breast tumor subtypes in independent gene expression data sets.. Proc Natl Acad Sci U S A.

[3] Furge KA, Lucas KA, Takahashi M, Sugimura J, Kort EJ (2004). Robust classification of renal cell carcinoma based on gene expression data and predicted cytogenetic profiles.. Cancer Res.

[4] Alizadeh AA, Eisen MB, Davis RE, Ma C, Lossos IS (2000). Distinct types of diffuse large B-cell lymphoma identified by gene expression profiling.. Nature.

[5] Segal E, Friedman N, Koller D, Regev A (2004). A module map showing conditional activity of expression modules in cancer.. Nat Genet.

[6] Hummel M, Bentink S, Berger H, Klapper W, Wessendorf S (2006). A biologic definition of burkitt's lymphoma from transcriptional and genomic profiling.. N Engl J Med.

[7] Taylor BS, Schultz N, Hieronymus H, Gopalan A, Xiao Y (2010). Integrative genomic profiling of human prostate cancer.. Cancer Cell.

[8] Shen R, Olshen AB, Ladanyi M (2009). Integrative clustering of multiple genomic data types using a joint latent variable model with application to breast and lung cancer subtype analysis.. Bioinformatics.

[9] Smolkin M, Ghosh D (2003). Cluster stability scores for microarray data in cancer studies.. BMC Bioinformatics.

[10] Antoniak C (1974). Mixtures of Dirichlet processes with applications to Bayesian nonparametric problems.. Ann Stat.

[11] Ferguson T (1973). A Bayesian analysis of some nonparametric problems.. Ann Stat.

[12] Savage RS, Ghahramani Z, Griffin JE, de la Cruz B (2010). Discovering transcriptional modules by bayesian data integration.. Bioinformatics.

[13] Kundaje A, Middendorf M, Gao F, Wiggins C, Leslie C (2005). Combining sequence and time series expression data to learn transcriptional modules.. IEEE/ACM Trans Comput Biol Bioinform.

[14] Berger JA, Hautaniemi S, Mitra SK, Astola J (2006). Jointly analyzing gene expression and copy number data in breast cancer using data reduction models.. IEEE/ACM Trans Comput Biol Bioinform.

[15] Chin S, Teschendorff A, Marioni J, Wang Y, Barbosa-Morais N (2007). High-resolution acgh and expression profiling identifies a novel genomic subtype of er negative breast cancer.. Genome Biol.

[16] Chin K, Devries S, Fridlyand J, Spellman PT, Roydasgupta R (2006). Genomic and transcriptional aberrations linked to breast cancer pathophysiologies.. Cancer Cell.

[17] Jiang M, Li M, Fu X, Huang Y, Qian H (2008). Simultaneously detection of genomic and expression alterations in prostate cancer using cdna microarray.. Prostate.

[18] Rasmussen CE (2000). The infinite Gaussian mixture model.. Proceedings of Advances in Neural InformationProcessing Systems 12.

[19] Wild D, Rasmussen C, Ghahramani Z, Cregg J, de la Cruz BJ (2002). A Bayesian approach to modeling uncertainty in gene expression clusters.. Proceedings of 3rd International Conference on Systems Biology, Sweden.

[20] Medvedovic M, Sivaganesan S (2002). Bayesian infinite mixture model based clustering of gene expression profiles.. Bioinformatics.

[21] Medvedovic M, Yeung KY, Bumgarner RE (2004). Bayesian mixture model based clustering of replicated microarray data.. Bioinformatics.

[22] Liu X, Sivaganesan S, Yeung KY, Guo J, Bumgarner RE (2006). Context-specific infinite mixtures for clustering gene expression profiles across diverse microarray dataset.. Bioinformatics.

[23] Dahl D, Kim- Anh Do MVE Peter Müller, editor (2006). Model-based clustering for expression data via a Dirichlet process mixture model.. Bayesian Inference for Gene Expression and Proteomics.

[24] Qin ZS (2006). Clustering microarray gene expression data using weighted Chinese restaurant process.. Bioinformatics.

[25] Rasmussen C, de la Cruz B, Ghahramani Z, Wild DL (2007). Modeling and visualizing uncertainty in gene expression clusters using Dirichlet process mixtures.. IEEE/ACM Trans Comput Biol Bioinform.

[26] van de Wiel MA, van Wieringen WN (2007). Cghregions: Dimension reduction for array cgh data with minimal information loss.. Cancer informatics.

[27] Smyth GK (2005). Limma: linear models for microarray data.. Bioinformatics and Computational Biology Solutions using R and Bioconductor.

[28] Prasad, Goel R, Kandasamy K, Keerthikumar S, Kumar S (2009). Human Protein Reference Database–2009 update.. Nucleic Acids Res.

[29] Beisser D, Klau GW, Dandekar T, Müller T, Dittrich MT (2010). BioNet: an R-Package for the functional analysis of biological networks.. Bioinformatics.

[30] Sieuwerts AM, Look MP, Meijer-van Gelder ME, Timmermans M, Trapman AM (2006). Which cyclin e prevails as prognostic marker for breast cancer? results from a retrospective study involving 635 lymph node negative breast cancer patients.. Clin Cancer Res.

[31] Frescas D, Pagano M (2008). Deregulated proteolysis by the F-box proteins SKP2 and TrCP: tipping the scales of cancer.. Nat Rev Cancer.

[32] Langerod A, Zhao H, Borgan O, Nesland J, Bukholm I (2007). Tp53 mutation status and gene expression profiles are powerful prognostic markers of breast cancer.. Breast Cancer Res.

[33] Kanehisa M, Araki M, Goto S, Hattori M, Hirakawa M (2008). KEGG for linking genomes to life and the environment.. Nucleic Acids Res.

[34] Merico D, Isserlin R, Stueker O, Emili A, Bader GD (2010). Enrichment map: A network-based method for gene-set enrichment visualization and interpretation.. PLoS ONE.

[35] Wang X, Terfve C, Rose JC, Markowetz F (2011). HTSanalyzeR: a R/Bioconductor package for integrated network analysis of high-throughput screens.. Bioinformatics.

[36] Ertel A, Verghese A, Byers SW, Ochs M, Tozeren A (2006). Pathway-specific differences between tumor cell lines and normal and tumor tissue cells.. Mol Cancer.

[37] Miecznikowski J, Wang D, Liu S, Sucheston L, Gold D (2010). Comparative survival analysis of breast cancer microarray studies identifies important prognostic genetic pathways.. BMC Cancer.

[38] Rubin JB (2009). Chemokine signaling in cancer: One hump or two?. Semin Cancer Biol.

[39] Hembruff SL, Cheng N (2009). Chemokine signaling in cancer: Implications on the tumor microenvironment and therapeutic targeting.. Cancer Ther.

[40] Thurn KT, Arora H, Paunesku T, Wu A, Brown EMB (2011). Endocytosis of titanium dioxide nanoparticles in prostate cancer pc-3m cells.. Nanomedicine.

[41] Polo S, Pece S, Di Fiore PP (2004). Endocytosis and cancer.. Curr Opin Cell Biol.

[42] Zheng C, Ren Z, Wang H, Zhang W, Kalvakolanu DV (2009). E2f1 induces tumor cell survival via nuclear factor-kappab-dependent induction of egr1 transcription in prostate cancer cells.. Cancer Res.

[43] van deWiel M, Kim K, Vosse S, vanWieringen W, Wilting S (2007). CGHcall: calling aberrations for array CGH tumor profiles.. Bioinformatics.

[44] Geier F, Timmer J, Fleck C (2007). Reconstructing gene-regulatory networks from time series, knock-out data, and prior knowledge.. BMC Syst Biol.

[45] Warnat P, Eils R, Brors B (2005). Cross-platform analysis of cancer microarray data improves gene expression based classification of phenotypes.. BMC Bioinformatics.

[46] Bicciato S, Spinelli R, Zampieri M, Mangano E, Ferrari F (2009). A computational procedure to identify significant overlap of differentially expressed and genomic imbalanced regions in cancer datasets.. Nucleic Acids Res.

